# Chemical and Structural Responses to Downregulated *p*-Hydroxycinnamoyl-Coenzyme A: Quinate/Shikimate *p*-Hydroxycinnamoyltransferase in Poplar Cell Walls

**DOI:** 10.3389/fpls.2021.679230

**Published:** 2022-01-25

**Authors:** Minglei Su, Yingli Liu, Jianxiong Lyu, Shutang Zhao, Yurong Wang

**Affiliations:** ^1^Research Institute of Wood Industry, Chinese Academy of Forestry, Beijing, China; ^2^Key Laboratory of National Forestry and Grassland Administration/Beijing for Bamboo and Rattan Science and Technology, International Centre for Bamboo and Rattan, Beijing, China; ^3^State Key Laboratory of Tree Genetics and Breeding, Research Institute of Forestry, Chinese Academy of Forestry, Beijing, China

**Keywords:** poplar, *HCT*, lignin, cell wall components, structural properties

## Abstract

Unraveling the impact of lignin reduction on cell wall construction of poplar stems is important for accurate understanding the regulatory role of biosynthetic genes. However, few cell-level studies have been conducted on the changes in lignin, other important cell wall composition, and the structural properties of transgenic poplar stems at different developmental stages. In this work, the content and microdistributions of cell wall composition as well as the morphological characteristics of cells were studied for *p*-hydroxycinnamoyl-coenzyme A:quinate/shikimate *p*-hydroxycinnamoyltransferase (*HCT*) downregulated transgenic poplar 84K (*Populus alba × P. glandulosa cl. ‘84k’*) at different developmental stages. Results show that the lignin contents of the upper, middle, and basal parts of *HCT* transgenic poplar stems were significantly decreased by 10.84, 7.40, and 7.75%, respectively; and the cellulose contents increased by 8.20, 6.45, and 3.31%, respectively, compared with the control group. The cellulose/lignin ratio of *HCT* transgenic poplars was therefore increased, especially in the upper sections, where it was 23.2% higher. Raman results indicate the appearance of *p*-hydroxyphenyl units (H) and a decrease in the ratio of syringyl/guaiacyl (S/G) lignin monomers in fiber cell walls of *HCT* transgenic poplars. In addition, microstructure observations revealed that the fiber and vessel cells of the *HCT* transgenic poplars exhibited thin cell walls and large lumen diameters. Compared with the control group, the cell wall thickness of fiber and vessel cells decreased by 6.50 and 10.93% on average, respectively. There was a 13.6% decrease in the average ratio of the cell wall thickness to the lumen diameter and an increase in fiber length and width of 5.60 and 6.11%, respectively. In addition, downregulation of *HCT* did not change the orientation of cellulosic microfibrils, but it led to an 11.1% increase of the cellulose crystallinity in cell walls compared to the control poplars. The information obtained herein could lead to a better understanding of the effects of genetic modifications on wood cell walls.

## Introduction

Lignocellulosic material is poised to be the primary source for biorefinery processes, and it can be used to produce liquid biofuels, chemicals, and materials (Vanholme et al., 2013). Wood is the main source of lignocellulosic biomass for the production of liquid biofuels, which consists of carbohydrates (including cellulose and hemicellulose) and lignin. However, the presence of lignin in plant cell walls is one of the most important reasons for biomass recalcitrance and the removal of lignin is costly and environmentally damaging because of the use of chemicals (Hu et al., 1999; Pauly and Keegstra, 2008). A high proportion of syringyl/guaiacyl (S/G) lignin monomers in wood can greatly improve biorefining efficiency (Reddy et al., 2005). Thus, lignin genetic engineering is considered to be a promising route to improve enzymatic digestion and delignification efficiency by reducing the lignin content or changing the lignin monomeric composition (Vanholme et al., 2010, 2012).

The *p*-hydroxycinnamoyl-coenzyme A:quinate/shikimate *p*-hydroxycinnamoyltransferase (*HCT*) is on the upstream and downstream in the phenylpropanoid pathway of *p*-coumarate 3′-hydroxylase (*C3′H*), and co-catalysis with *C3′H* of the conversion process from *p*-coumaroyl coenzyme A to caffeoyl coenzyme A (Hoffmann, 2002; Ralph et al., 2012). Previous studies have found that *HCT* gene silencing resulted in depression of lignin content and changes in lignin monomeric composition in *Arabidopsis* and *Pinus radiata* (Hoffmann et al., 2004; Wagner et al., 2007). It has also been found that downregulation of the *C3′H* and *HCT* in alfalfa had a significant effect on the structure of ball-milled lignin (Pu et al., 2009). Traditional studies of lignin content and monolignol composition have tended to use Klason lignin and nuclear magnetic resonance (NMR) spectroscopy analysis. However, the obtained native lignin samples for structural characterization are usually pretreated by physical and chemical methods, which may alter the natural molecular structure of lignin (Ralph et al., 2012; Peng et al., 2014). Therefore, further *in situ* studies on the lignin monomeric composition and its distribution in transgenic poplars are needed. With the development of Raman technology, the attribution peaks of G, S, and H monomers in Raman spectra are becoming clearer (Saariaho et al., 2003; Jin et al., 2018). This article uses Raman technology to observe the changes in lignin monomers in different regions of *HCT* transgenic poplar cell wall.

The downregulation of lignin biosynthetic genes not only causes changes in cell wall components, but also causes morphological changes in cells, which leads to plant growth phenotypic changes (Jung et al., 2012; Wang et al., 2014; Zhang et al., 2014). For example, when *C3′H* and *HCT* were simultaneously downregulated in alfalfa, the plants could not continue to grow upright beyond a certain height, and plant cell walls became thinner (Tong et al., 2015). When downregulating *HCT* in poplars, the thickness of their fiber and vessel cell walls have been shown to decrease (Zhou et al., 2018). However, studies are lacking regarding the effect of downregulation of *HCT* on the chemical composition and morphology of cell walls in *HCT* transgenic poplars and other transgenic plants at different developmental stages of stems.

The ultrastructure of wood fiber cell walls has an important influence on the physical and mechanical properties of wood. Indeed, the alteration of lignin content and composition in transgenic plants have been observed to affect the ultrastructure of fiber cell walls. In both *Arabidopsis* and tobacco limited in cinnamoyl-CoA reductase *(CCR)* activity, disordered cellulose microfibril organization was observed in fibers (Ruel et al., 2001, 2009). However, studies on the ultrastructure of *HCT* transgenic poplars have not been reported. Tracking the changes in cell wall structure, crystallinity, and microfibril angle (MFA) of transgenic poplar is essential to understand the responses of wood cell walls to downregulation of *HCT*.

In this study, we conducted a comprehensive study on *HCT* downregulated poplars using multiple techniques, combining chemical and structural analyses. The lignin and cellulose content and micro-region distribution of *HCT* transgenic poplars stems at different developmental stages, as well as the lignin monomers were observed to understand the responses of cell walls’ chemical properties to downregulated *HCT*. Further, structural properties, such as fiber and vessel cell thickness and lumen diameter, fiber length and width, cell wall ultrastructure, cellulose crystallinity, and MFA of *HCT* transgenic poplars were obtained.

## Materials and Methods

### Plant Materials

**FIGURE 1:**
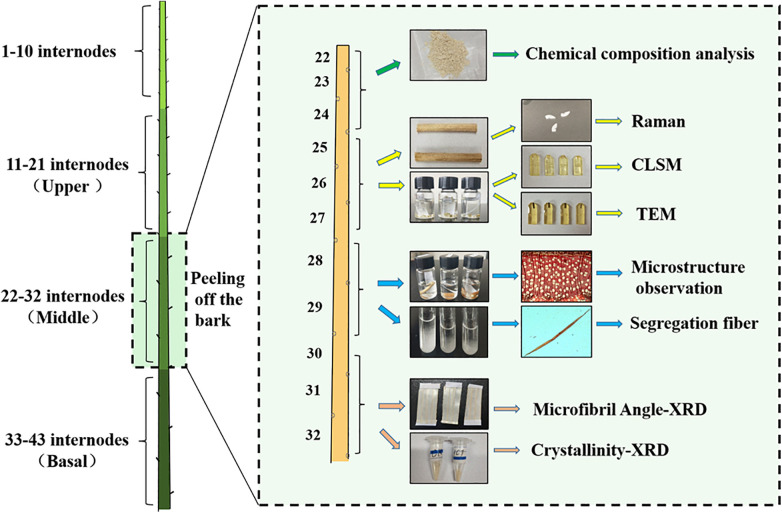
Sampling and preservation from poplar stems; the preparation of wood samples from the middle parts for chemical and structural analyses is illustrated.

The tested specimens were prepared from control 84K (CK) poplars (*Populus alba × P. glandulosa cl.*) and downregulated *HCT* transgenic poplars. A RNAi construct for downregulating *HCT* gene (Orthologous of Potri.001G042900) expression in poplar was made in the pBIRNAi vector and transformed into poplars previously (Peng et al., 2014). Transgenic poplars with significantly reduced *HCT* gene expression were obtained, and the expression of the *HCT* was reduced by more than 65% compared with the control poplars. The CK and *HCT* transgenic poplars were planted in a greenhouse with 16 h natural light during the daytime (22°C) and 8 h of darkness at night (15°C). Poplar trees (98–124 cm height) were harvested after one growth season (6 months). The stem diameter of *HCT* transgenic poplars (4.72 mm) was thinner than that of CK poplars (5.25 mm). The sampling process for transgenic and CK poplars is shown in Figure 1. The leaves were manually removed from the poplars’ stems and cut into three parts from the 11 internodes to the base. Next, some of the samples obtained from the upper, middle, and basal parts were preserved in FAA (70% alcohol: formalin: acetic acid = 90:5:5) fixative and 2.5% glutaraldehyde; some samples were packed in foil and placed in a refrigerator for subsequent structural and ultrastructural observation. The remaining stems were air-dried and prepared for analysis of cell wall composition. Sample preparation of the middle parts is illustrated in Figure 1.

### Cell Wall Composition Analyses

The samples from upper, middle, and basal parts of the CK and *HCT* transgenic poplar stems were selected from the air-dried materials and treated with alcohol to obtain insoluble residues. The monosaccharide composition was determined using gas chromatograph mass spectrometry (Agilent) as described previously (Zhang and Zhou, 2017). For cellulose contents, the remains obtained above after trifluoroacetic acid treatment are hydrolyzed with sulfuric acid. After development of color with anthrone in concentrated sulfuric acid, the absorption at 625 nm is measured and the contents are determined based on standard curves (Zhang and Zhou, 2017). The lignin content of the wood was determined using the acetyl bromide method as previously reported (Foster et al., 2010). Five biological replicates were taken to ensure the accuracy of the results.

### Confocal Laser Scanning Microscopy Analysis

The samples of the upper, middle, and basal parts of the CK and *HCT* transgenic poplars were taken from 2.5% glutaraldehyde and prepared at sizes of 1 mm × 1 mm × 3 mm. The samples were washed with 0.1 M phosphate-buffered saline (PBS) and deionized water three times (10 min each wash), then dehydrated by gradient alcohol. Next, the ethanol was replaced by propylene oxide gradually. The samples were then permeated in propylene oxide and Epon812 resin at ratios of 2:1, 1:1, and 1:2 three times, and placed in vacuum for 1.5–2 h. Finally, the embedded samples were put in vacuum and polymerized for 12 h at 37°C, 12 h at 45°C, and for 24 h at 60°C, respectively. Serial of 1.5-μm-thick cross-sections were cut from samples using an ultramicrotome (Leica EMUC6, Wetzlar, Germany), stained with acridine orange solution (0.001%) for 10 min, mounted with 70% glycerol, and then observed using a confocal laser scanning microscope (ZEISS LSM510META). A 488-nm argon laser was used for excitation.

### Confocal Raman Microscopy Analysis

The samples for Raman analysis were taken from the air-dried stems. Serial of 16-μm-thick cross-sections were cut using a sliding microtome (Leica SM 2010R, Wetzlar, Germany) from the three parts of CK and transgenic poplars for Raman analysis. A LabRam Xplora exquisite full-automatic confocal Raman microscope (Horiba Jobin Yvon, Paris, France) equipped with an MPlan 100× oil immersion microscope objective (Olympus, NA = 1.40) was utilized. A linear polarized laser (diode-pumped green laser, λ = 532 nm), focused with a diffraction-limited spot size (0.61λ/NA), was used to conduct measurements. The laser power on the sample was approximately 8 mW. For mapping, an integration time of 2 s was chosen and every pixel corresponds to one scan with a spectrum acquired every 0.4 μm by averaging 2 s cycles. The Spectrum 6 software package was used for spectra analysis and image processing.

### Observation of Anatomic Construction

The samples from three different parts of CK and transgenic poplars stems were taken from FAA fixative. Slices with a thickness of 20 μm were prepared using a sliding microtome. The samples were stained with safranine (2%) for 12 h and washed with deionized water for three times (10 min for each), then a graded ethanol series was carried out. Finally, permanent sections were made with resin, which was used to observe the microstructure and measure the morphological parameters of fibrous cells. The ZEISS Imager A1 light microscope and Axiovision image processing software were used. Hundred fibers and 100 vessels were selected to measure cell wall thickness and lumen diameter.

### Determination of Fiber Length and Width

The wood samples with a size of 1 mm × 1 mm × 10 mm (R × T × L) were cut from air-dried stems of three parts in the CK and transgenic poplars. Then, the samples were put into a centrifuge tube and 30 ml of dialysis solution was added (40% hydrogen peroxide: glacial acetic acid: water = 4:5:21) before heating in an oven (80°C) for 3 days. The isolated samples were cleaned three times with deionized water. After staining with safranine, the samples were observed under the ZEISS Imager A1 light microscope and images of the samples were taken. Hundred fibers were selected and their lengths and widths were measured using Axiovision image processing software.

### Ultrastructure Observation

The samples of the middle part of poplar stems were obtained from 2.5% glutaraldehyde and cut into 1 mm^3^ blocks for ultrastructure observation. The tissues were washed with 0.1 M PBS (1 h each wash) and deionized water three times (10 min each wash), then fixed in 1% osmium acid solution at 4°C. After 8 h, the samples were washed with PBS and water, then dehydrated and embedded in Epon812 resin. The embedding process is the same as that for confocal laser scanning microscopy analysis (CLSM) samples above. Transverse, ultrathin sections were cut with a 70 nm thickness using an ultramicrotome (Leica EMUC6, Wetzlar, Germany). Observations were carried out with a transmission electron microscope (TEM) (HT7700, Hitachi, Japan) at 100 kV accelerating voltage.

### Measurements of Microfibril Angle

The slices with a 100 μm thickness were used for MFA analysis, which were cut from the middle and basal parts of the air-dried stems in *HCT* transgenic and control poplars. The MFAs were measured by an X’pert PRO polycrystalline X-ray diffractometer (PANalytical Company, Netherlands). The basic parameters of the diffractometer are as follows: diffraction angle = 22.4°, rotation range = 0–360°, rotation step = 0.5°, tube voltage = 40 kV, and tube current = 40 Ma. After rotating 360°, the 002 plane diffraction pattern was obtained. The average MFA was calculated by the 0.6T method (Wang et al., 2012). Three replicates of each sample were carried out.

### Determination of Crystallinity

The wood powers from middle and basal parts of the air-dried stems in *HCT* transgenic and control poplars were used for determination of crystallinity. A D/max-rB type rotating anode X-ray diffractometer (Rigaku Company, Japan) was used for this purpose. The operating parameters for the measurements are as follows: radiation tube voltage = 40 kV, scanning range = 5°–40°, step length = 0.02°, and scanning speed = 4°/min. The crystallinity was calculated for the *HCT* transgenic and CK poplars by the Segal method, based on the diffraction patterns. The crystallinity equation is C_r_I = (*I*_*u*_ − *I*_*a*_)/*I*_*u*_ × 100%, where *I*_*u*_ is the maximum integral intensity of wood fiber diffraction intensity at 2θ = 22° and *I*_*a*_ represents the minimum integral intensity at 2θ = 18°. Three replicates of each sample were carried out.

## Results

### Chemical Changes of Cell Wall in Hydroxycinnamoyltransferase Transgenic Poplars

The contents and microdistributions of cell wall composition of the *HCT* transgenic poplars xylem at different developmental stages were studied to understand wood cell wall responses to downregulated *HCT*.

#### Chemical Composition Changes of Cell Wall

The lignin, cellulose, and hemicellulose content were determined from the stems of poplars at different developmental stages. Compared with the CK poplars, the lignin content of *HCT* transgenic poplars was significantly decreased by 10.84, 7.40, and 7.75% in the upper, middle, and basal parts, respectively (Table 1). And there was a significant increase in cellulose content in the upper and middle parts of transgenic poplars, by 8.20 and 6.45%, respectively. In comparison, the cellulose content of the basal part increased by only 3.31%. The cellulose/lignin ratio of *HCT* transgenic poplar increased (Table 1), and the increasing of upper parts was higher (23.2%) than those of the middle and basal sections (increased by 11.2 and 11.8%). The content of hemicelluloses (depicted by the cell wall-related monosaccharides rhamnose, fucose, arabinose, xylose, mannose, and galactose) does not change significantly in upper and basal part of *HCT* transgenic plants. But the hemicelluloses content in middle part significant decreased compared with the CK, and significant changes in the content of almost all cell wall-related monosaccharides. In which, the rhamnose, fucose, arabinose, and xylose content decreased and the mannose, galactose, and glucose content increased. In addition, the *HCT* transgenic poplars had higher mannose content in all three parts of stems.

**TABLE 1:** Chemical composition of *HCT* transgenic and CK poplar cell wall at different developmental stages.

Composition	CK (μg/mg)			*HCT* (μg/mg)		
	Upper	Middle	Basal	Upper	Middle	Basal
Lignin	161 (±3.3)^B^	158 (±4.7)^B^	159 (±2.5)^B^	141 (±4.6)^A^	151 (±6.4)^AB^	146 (±3.3)^A^
Cellulose	386 (±15.4)^A^	423 (±13.0)^BC^	430 (±5.1)^BCD^	418 (±11.2)^B^	450 (±19.8)^D^	444 (±8.0)^CD^
Hemicellulose	250 (±11.0)^B^	249 (±11.7)^B^	244 (±10.3)^B^	247 (±2.69)^B^	225 (± 10.4)^A^	234 (±8.67)^AB^
Cellulose/lignin	2.41	2.68	2.71	2.97	2.98	3.03
Rhamnose	5.39 (±0.25)^B^	5.26 (±0.28)^B^	4.73 (±0.34)^A^	5.51 (±0.29)^B^	4.60 (±0.24)^A^	4.65 (±0.07)^A^
Fucose	1.15 (±0.04)^BC^	1.10 (±0.07)^B^	1.03 (±0.07)^A^	1.20 (±0.01)^C^	1.02 (±0.04)^A^	1.01 (±0.03)^A^
Arabinose	4.45 (±0.14)^D^	3.82 (±0.18)^C^	3.44 (±0.23)^B^	4.53 (±0.12)^D^	3.30 (±0.13)^AB^	3.15 (±0.09)^A^
Xylose	220 (±10.8)^C^	220 (±9.1)^C^	215 (±9.7)^BC^	214 (±9.3)^BC^	194 (±9.3)^A^	204 (±2.6)^AB^
Mannose	10.3 (±0.54)^A^	11.6 (±0.66)^B^	12.7 (±0.34)^C^	12.2 (±0.53)^BC^	14.0 (±0.51)^D^	13.8 (±0.64)^D^
Galactose	8.50 (±0.45)^B^	7.98 (±0.34)^B^	7.22 (±0.62)^A^	9.23 (±0.36)^C^	8.01 (±0.25)^B^	7.15 (±0.30)^A^
Glucose	49.2 (±3.66)^A^	56.6 (±2.97)^B^	54.9 (±2.89)^B^	54.0 (±2.38)^B^	64.6 (±3.52)^C^	55.8 (±2.42)^B^

*All values are expressed as means ± SD (n = 5, biological replicates). A, B, C, and D in the table indicated multiple analysis results, the same letter meant that there were no significant differences, and the different letters meant there were significant differences, p < 0.05 (Student Newman–Keuls test).*

The poplars at different stages of development showed some variation in their cell wall composition. Results revealed that more hemicellulose was deposited in younger internodes (upper part) and more cellulose was present in older internodes (middle and basal parts) for the transgenic and CK poplars. After the downregulation of *HCT*, poplars show more reduced lignin and increased cellulose in younger internodes.

#### Distribution of Lignin in the Xylem Cell

**FIGURE 2:**
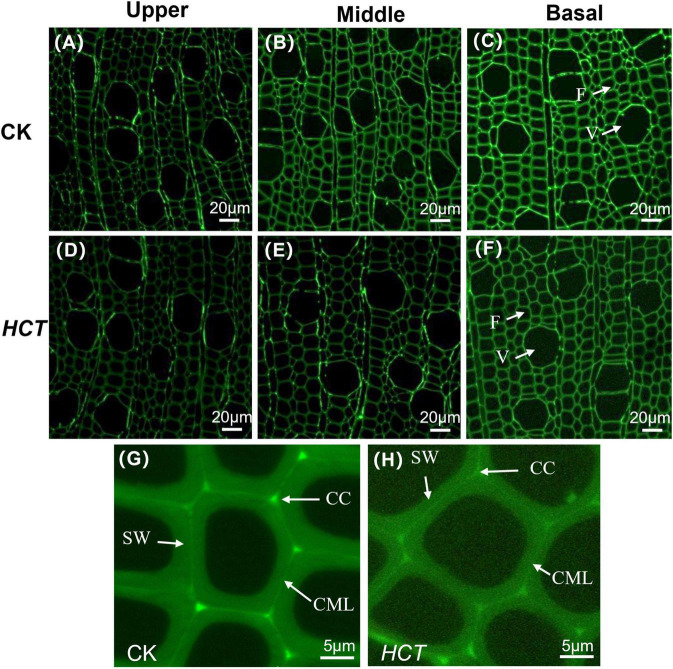
Microscopic images of *HCT* transgenic and CK poplars stems by CLSM at different developmental stages. CK poplar, **(A)** the upper part; **(B)** the middle part; **(C)** the basal part; *HCT* transgenic poplar, **(D)** the upper part; **(E)** the middle part; **(F)** the basal part. Bar = 20μm. **(G)** The magnified image of CK poplar; **(H)** the magnified image of *HCT* transgenic poplar. Bar = 5 μm. F, fiber; V, vessel.

As can be seen from Figure 2, CLSM images were obtained in a large field of view for the lignin distribution in poplar cross sections. The cell wall corner (CC) and the compound middle lamella (CML) regions have stronger fluorescence intensity, indicating that these regions had a higher degree of lignification. Compared with the CK poplars, a lower fluorescence intensity was observed clearly in the upper, middle, and basal parts of the *HCT* transgenic poplars (Figures 2D–F). Besides, the intensity in the CC, CML, and the secondary wall (SW) region of the *HCT* transgenic poplars were all decreased when compared with those of CK (Figures 2G,H). This indicated that the downregulation of *HCT* reduced the lignin concentration, which is coincident with the findings from cell wall composition analysis. The downregulation of *HCT* did not change the characteristics of lignin distribution, whose content remained CC > CML > SW. At different developmental stages of poplar stems, lignin concentrations increased gradually with the degree of lignification.

#### Distribution of Lignin and Cellulose in Wood Cell Wall

**FIGURE 3:**
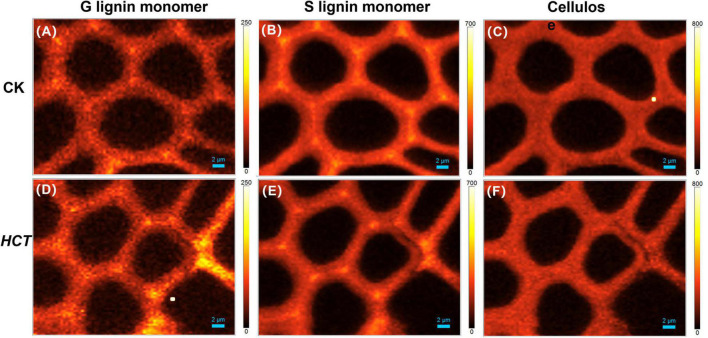
Raman images of the lignin distributions for *HCT* transgenic and CK poplars at different developmental stages. CK poplar, **(A)** the upper part; **(B)** the middle part; **(C)** the basal part; *HCT* transgenic poplar, **(D)** the upper part; **(E)** the middle part; **(F)** the basal part. Bar = 2 μm. The lignin was integrated from 1550 to 1700 cm^–1^.

Raman spectroscopy technology can clearly visualize the distribution of lignin, lignin monomers, and carbohydrates in poplar cell wall *in situ*. Raman spectral characteristic peaks and their attribution of cell wall composition are listed in supporting Table 2. Typical Raman images of lignin are shown in Figure 3 for the CK and *HCT* transgenic poplars at different developmental stages by calculating the Raman band ranges from 1550 to 1700 cm^–1^. High lignin concentration was visualized in CC and then CML; SW was the lowest for both the *HCT* transgenic and CK poplars. The intensities in the upper, middle, and basal parts of the cell wall in the *HCT* transgenic poplars were all lower than those in CK poplars, due to the decline of lignin concentration. At different developmental stages of poplar stems, lignin concentrations increased gradually with the degree of lignification.

**TABLE 2:** Raman spectral characteristic peaks and their attribution of cell wall composition.

Composition	Wavenumber/cm^–1^	Band assignments
Lignin	1272	Aryl-O of aryl-OH and aryl-O-CH_3_ of the G lignin
	1332	Aryl-O-CH_3_ vibration of the S lignin
	1216	Aryl-O of aryl-OH and aryl-O-CH_3_, ring deformation of the H lignin
	1600	Symmetric aryl ring stretching
	1654	Ring-conjugated C = C stretch of coniferyl alcohol; C = O stretch of conifer aldehyde
Cellulose	377	C-C-C ring deformation vibration, heavy atom stretching cellulose I
Cellulose and hemicellulose	1095 1122	Asymmetric stretch of C-O-C linkages Symmetric stretch of C-O-C linkages
Cellulose and lignin	1460	CH_3_ bending in the O-CH_3_ group, CH_2_ scissoring

*According to the literatures Wiley and Atalla (1987), Schenzel and Fischer (2001), Saariaho et al. (2003), Agarwal (2006), Agarwal and Ralph (2008), Kanbayashi and Miyafuji (2015), and Jin et al. (2018).*

**FIGURE 4:**
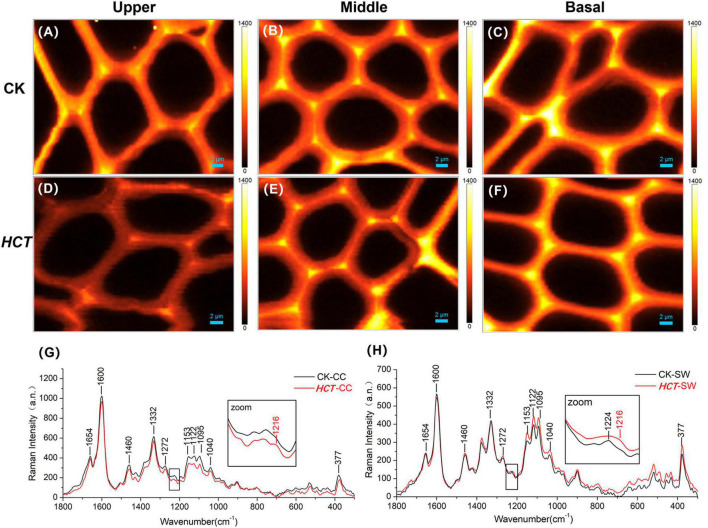
Raman images of the lignin monomers and cellulose distributions, and extracted average spectra in the middle parts of *HCT* transgenic and CK poplars. CK poplar, **(A)** G lignin monomer; **(B)** S lignin monomer; **(C)** cellulose; *HCT* transgenic poplar, **(D)** G lignin monomer; **(E)** S lignin monomer; **(F)** cellulose. Bar = 2 μm. **(G)** Raman spectra obtained from the CC regions in cell wall; **(H)** Raman spectra obtained from the SW regions in cell wall. G lignin monomer integrating from 1247 to 1289 cm^–1^; S lignin monomer from 1306 to 1359 cm^–1^; cellulose from 344 to 400 cm^–1^. The spectra of the transgenic lines exhibit a shoulder at around 1216 cm^–1^ (assigned to aryl-O of aryl-OH and aryl-O-CH_3_ from H lignin).

The middle part of poplars was used as tested specimen to study the distribution of lignin monomer and cellulose in wood cell walls. And average Raman spectra in the region of 300–1800 cm^–1^ of CC and SW were obtained (Figure 4). Stretching vibrations at 1600 and 1654 cm^–1^ were assigned to the lignin. Additionally, the results from previous studies reported that the peaks of G, S, and H lignin monomer in poplars were verified at 1272, 1333, and 1216 cm^–1^, respectively (Saariaho et al., 2003; Agarwal et al., 2011; Jin et al., 2018). Although the spectra of *HCT* plants tended to be similar to those of the CK, the lignin spectrum intensities of CC and SW of the *HCT* transgenic poplars at 1600 cm^–1^ were lower than those of CK poplars. As shown in the Raman images, there were no obvious changes in the G lignin monomer of the transgenic and CK poplars. However, the S lignin monomer in CK poplars occurred at a higher intensity than *HCT* poplar at SW regions, suggesting a decrease of S lignin monomer in the secondary cell walls of the *HCT* transgenic poplar, which was also observed in Raman spectra (Figure 4H). But no obvious difference was found about microdistribution of cellulose, both at Raman images (Figures 4C,F) and spectra (Figures 4G,H). Notably, the CC of *HCT* transgenic poplars was recorded with higher intensities at 1216 cm^–1^, which was assigned to the H lignin monomer (Figure 4).

#### Semiquantitative Analysis of Cellulose/Lignin and S/G Ratio

To extend the cell wall composition changes of cellulose/lignin and S/G ratio in *HCT* transgenic poplars at the cell level, a semiquantitative investigation was conducted. The ratio of I_377_/I_1600_ and I_1332_/I_1272_ represent the cellulose/lignin and S/G ratio, respectively. As illustrated in Table 3, the I_377_/I_1600_ (cellulose/lignin) ratio is higher in the SW region of *HCT* transgenic fibers, which is consistent with the results of the traditional chemical method (Table 1). However, no significant difference was found in the CC region, where the lignin concentration was high. Notably, the I_1332_/I_1272_ (S/G) ratio is lower both at the CC and SW regions in *HCT* transgenic fibers than those of the control, indicating that downregulation of *HCT* in poplars indeed decreased the S/G lignin monomer ratios at different regions of the cell wall.

**TABLE 3:** Relative intensity ratios of the cellulose/lignin and S/G lignin monomer in CC and SW regions of cell walls in the middle part of *HCT* transgenic and CK poplars.

Poplar	I_377_/I_1600_ (CC)	I_377_/I_1600_ (SW)	S/G = I_1332_/I_1276_ (CC)	S/G = I_1332_/I_1276_ (SW)
CK	0.20 (±0.02)	0.43 (±0.04)	4.37 (±0.48)	3.90 (±0.70)
*HCT*	0.20 (±0.02)	0.47 (±0.03)*	3.98 (±0.58)*	3.37 (±0.49)*

*All values are expressed as means ± SD (n = 7). Asterisk denotes significance at p < 0.1 (one-way ANOVA).*

### Structural Characteristics of Cell Wall in Hydroxycinnamoyltransferase Transgenic Poplars

The microstructure and ultrastructure characteristics of the *HCT* transgenic poplars were revealed to understand the response of structural properties of cell walls to downregulated *HCT*.

#### Morphology of Xylem Cell

**FIGURE 5:**
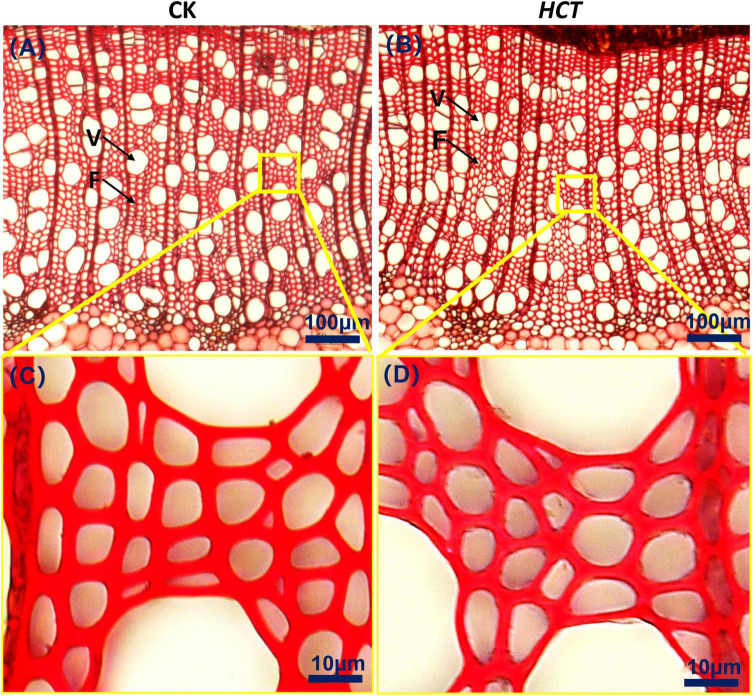
Cross-sectional microstructure images of upper parts in poplars. **(A)** CK poplar; **(B)** *HCT* transgenic poplar, Bar = 100 μm. **(C)** Enlarged version of **(A)**; **(D)** enlarged version of **(B)**, Bar = 10 μm. F, fiber; V, vessel.

From the images in Figures 5A,B, it can be discerned that the fibers of the two kinds of poplars are similar to each other. They are arranged regularly and distributed uniformly in the radial direction. The enlarged Figures 5C,D suggest that the fiber cell walls of *HCT* transgenic poplars are thinner. Fibers and vessels are the main cells of xylem in poplars, so the fiber and vessel cell thickness, lumen diameter and fiber length, and fiber width of the *HCT* transgenic poplars were measured.

#### Fiber Cell Thickness and Lumen Diameter

The fiber cell thickness and lumen diameter of the CK and *HCT* transgenic poplars are presented in Table 4. The average fiber cell wall thickness of the *HCT* transgenic poplars was 6.50% lower than that of the CK poplars. At different developmental stages of poplar stems, the cell wall thickness of the upper, middle, and basal parts of *HCT* transgenic poplars decreased by 6.47, 7.63, and 5.42%, respectively, and the fiber lumen diameter increased by 7.40, 11.88, and 5.45%, respectively, compared with the CK poplars. The ratio of the cell wall thickness to the lumen diameter of fiber cell walls is an important index for evaluating the quality of fiber cells. The results showed that the ratios in the upper, middle, and basal parts of transgenic poplars were all smaller than those of the control poplars in the corresponding parts and were reduced by 13.6% on average.

**TABLE 4:** Fiber cell parameters of *HCT* transgenic and CK poplars at different developmental stages.

Line	Parts	Cell wall thickness/μm	Lumen dimeter/μm	Cell wall thickness/lumen dimeter	Length/μm	Width/μm	Length/width
CK	Upper	2.32 (±0.211)^B^	9.59 (±2.01)^A^	0.242 (±0.023)^C^	446 (±72)^A^	18.0 (±4.51)^AB^	24.8 (±3.06)^A^
	Middle	2.36 (±0.306)^B^	10.1 (±1.46)^A^	0.233 (±0.031)^C^	467 (±69)^B^	17.4 (±2.29)^A^	26.5 (±4.00)^AB^
	Basal	2.40 (±0.256)^B^	11.0 (±2.15)^B^	0.218 (±0.037)^B^	480 (±79)^B^	18.4 (±2.73)^BC^	26.1 (±3.50)^AB^
*HCT*	Upper	2.17 (±0.244)^A^	10.3 (±1.10)^A^	0.210 (±0.028)^B^	469 (±72)^B^	17.9 (±2.65)^AB^	24.8 (±3.51)^A^
	Middle	2.18 (±0.360)^A^	11.3 (±3.24)^B^	0.193 (±0.027)^A^	486 (±101)^B^	19.2 (±2.55)^C^	25.3 (±4.47)^AB^
	Basal	2.27 (±0.364)^AB^	11.6 (±2.70)^B^	0.196 (±0.032)^A^	516 (±77)^C^	19.1 (±2.68)^C^	27.0 (±4.22)^B^

*All values are expressed as means ± SD (fiber cell wall thickness and cell lumen diameter, n = 100; fiber length and fiber width, n = 100). A, B, and C in the table indicated multiple analysis results, the same letter meant that there were no significant differences, and the different letters meant there were significant differences, p < 0.05 (Student Newman–Keuls test).*

#### Fiber Length and Width

To further reveal the microscopic morphological characteristics of transgenic poplars, the wood was dissociated, and the length and width of individual fibers were measured (Table 4). The average fiber length of the *HCT* transgenic poplars (490 μm) was 5.60% higher than that of the CK poplars (464 μm), and the average fiber width of the *HCT* poplars (19.1 μm) was 6.11% higher than that of the control poplars (18.0 μm). At different developmental stages of poplar stems, the average fiber lengths of the three parts of the *HCT* transgenic poplars were 5.16, 4.07, and 7.50% greater than those of the CK poplars, respectively. The fiber width of the three parts of *HCT* poplars increased by 5.00, 9.09, and 3.80%, respectively. However, there was no significant difference in the ratio of fiber length to width between the two types of poplars.

#### Vessel Cell Thickness and Lumen Diameter

Table 5 shows the vessel cell wall thickness and its lumen diameter of poplars at different developmental stages of poplar stems. Thinner cell walls and larger lumen diameters of vessel cell in *HCT* transgenic poplars were observed. Vessel cell thickness in the three parts of the *HCT* transgenic poplars was reduced by 9.84, 13.97, and 8.37%, respectively, which significantly decreased compared with the CK poplars (an average decrease of 10.93%). The vessel lumen diameters of the upper, middle, and basal parts of the transgenic poplars were 10.12, 8.12, and 13.4% larger than those of the control poplars, respectively, and with an average increase of 9.83%. The vessel numbers and vessel area proportions obtained using Image J are listed in Table 5. The vessel numbers of the *HCT* transgenic poplars were slightly higher than those of CK poplars, but the vessel area proportions were similar. At different developmental stages of poplar stems, the vessel numbers and the vessel area proportions of the upper part were higher than those of the middle and basal parts for both *HCT* transgenic and CK poplars.

**TABLE 5:** Vessel cell parameters of *HCT* transgenic and CK poplars at different developmental stages.

Line	Parts	Cell wall thickness/μm	Lumen dimeter/μm	Area proportions %	Number
CK	Upper	1.83 (±0.325)^B^	30.8 (±4.59)^A^	21.1 (±2.05)^C^	39.5 (±3.25)^C^
	Middle	1.86 (±0.255)^B^	30.4 (±5.36)^A^	17.7 (±1.98)^B^	26.4 (±4.63)^A^
	Basal	1.79 (±0.274)^B^	32.1 (±5.16)^AB^	18.0 (±2.02)^B^	24.5 (±2.19)^A^
*HCT*	Upper	1.65 (±0.242)^A^	33.3 (±3.45)^AB^	20.3 (±2.90)^C^	42.2 (±5.69)^C^
	Middle	1.60 (±0.152)^A^	35.1 (±4.81)^B^	16.1 (±2.60)^A^	27.6 (±6.15)^A^
	Basal	1.64 (±0.215)^A^	35.6 (±3.29)^B^	19.4 (±0.80)^C^	30.8 (±0.96)^B^

*All values are expressed as means ± SD (vessel cell wall thickness and lumen diameter, n = 100; vessel area proportions and vessel numbers, n = 3). A, B, and C in the table indicated multiple analysis results, the same letter meant that there were no significant differences, and the different letters meant there were significant differences, p < 0.05 (Student Newman–Keuls test).*

#### Ultrastructure of Wood Cell Wall

**FIGURE 6:**
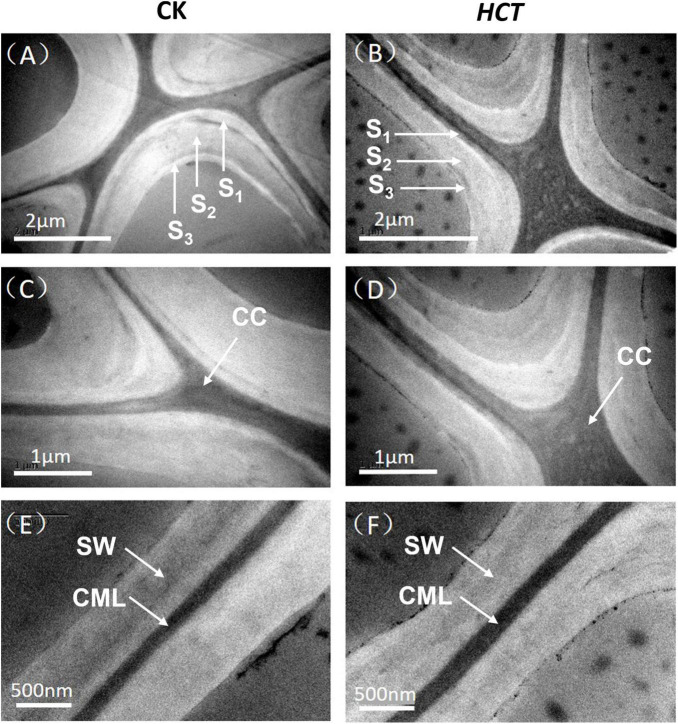
Transmission electron microscope images of the *HCT* transgenic and CK poplars. **(A)** The cell corner of CK poplar; **(B)** the cell corner of *HCT* transgenic poplar; **(C)** enlarged version of **(A)**; **(D)** enlarged version of **(B)**; **(E)** the cell wall of CK poplar; **(F)** the cell wall of *HCT* transgenic poplar.

In terms of cell wall layer structure, typical TEM images are shown in Figure 6 for the *HCT* transgenic and CK poplars. It can be seen that the morphology of the fiber cell walls was similar between the *HCT* transgenic and CK poplars. The intercellular layer and the secondary cell wall (contain S_1_, S_2_, and S_3_ layers) of the CK poplars could be observed clearly under TEM (Figures 6A,B). However, the cell wall layer structure of the transgenic poplars was less distinct, as shown in Figures 6C,D. The *HCT* transgenic poplar with thinner cell wall than that of the CK poplars is shown in Figures 6E,F. In addition, as can be seen in Figures 6D,F, the cell wall boundary of the transgenic poplars was not as smooth as that of the CK poplars.

The orientation of cellulose microfibrils in the SW and crystallinity of cellulose are the most important ultrastructural characteristics that determine the properties of wood and its products (Cave and Walker, 1994). Hence, average values of the MFA were obtained by using XRD and the 0.6T method. The average MFA of the *HCT* transgenic and CK poplars was 20.13° and 19.21°, respectively (Table 6), which coincide with typical values measured for juvenile wood (Gorisek and Niko, 1999). Although slightly higher, the MFA of transgenic lines is not significantly different from that of the CK. The *HCT* transgenic poplar showed an average relative crystallinity of 39.1%, whereas that of the CK had averaged crystallinity of 35.2%, according to Segal’s method (Table 6). The cellulose crystallinity of *HCT* transgenic poplars increased by 11.1% compared with that of the CK poplars, and this difference was statistically significant. This suggests that downregulation of *HCT* does not change the orientation of cellulosic microfibrils but increased the cellulose crystallinity of cell walls.

**TABLE 6:** Microfibril angle and cellulose crystallinity of *HCT* transgenic and CK poplars.

Sample	MFA^a^	CV^b^/%	Crystallinity/%	CV/%
CK	19.2 (±0.90)	4.70	35.2 (±1.17)	3.32
*HCT*	20.1 (±1.73)	8.61	39.1 (±0.20)*	5.21

*^a^Microfibril angle. ^b^Coefficient of variation. All values are expressed as means ± SD, except CV values (MFA, n = 6; crystallinity, n = 3). Asterisk denotes significance at p < 0.05 (one-way ANOVA).*

## Discussion

The reducing lignin content or changing lignin monomeric composition through breeding and genetic regulation has the potential to reduce costs in biomass processing such as pulp, paper, and lignocellulosic ethanol industries. Hitherto, the downregulation of *HCT* on the chemistry and structure at different developmental stages of poplar stems, as well as the ultrastructure of poplar trees had not been explored.

### Cell Wall Composition

The poplar cell walls are primarily composed of lignin, cellulose, and hemicellulose. In this study, we found that the downregulation of *HCT* led to a reduction in the lignin content and an increase in cellulose, as well as changes in monosaccharide content. A previous study exploring changes in the cellulose and hemicellulose contents of *HCT*-*C3′H* co-downregulated alfalfa, yielded similar results (Tong et al., 2015). The phenomenon of reduced lignin content accompanied by increased cellulose content has also been reported for hybrid poplars (Coleman et al., 2008) and aspens (*Populus tremuloides* Michx.) (Hu et al., 1999). This balancing mechanism between lignin and cellulose biosynthesis can be interpreted as the adaptability of trees to environmental change (Hu et al., 1999). To maintain the mechanical strength of lignin-deficient cell walls, plants may contribute to the conversion of glycosidic esters to cellulose and structural components (Tong et al., 2015). The production of fuel ethanol mainly uses cellulose and hemicellulose, so a higher cellulose/lignin ratio in *HCT* transgenic poplars facilitates the hydrolysis and fermentation process, increasing yields and ultimately reducing costs.

The composition of lignin, cellulose, and hemicellulose varied in poplars at different developmental stages. Lignin formation and deposition in cell walls is development tally controlled and reflects changes in gene expression. The results herein showed that lignin decreased more and cellulose increased in young internodes (upper) of poplar after downregulation of *HCT*. This is mainly because the basal part is the oldest internode, which is already highly lignified. Younger internodes, where lignification is still actively taking place, have high enzymatic activity and high levels of *HCT* gene repression expression (Chen et al., 2002).

### Lignin Structure

The reduced lignin concentration and/or altered monolignol composition have been studied extensively to reduce the recalcitrance of cell walls in biomass crops to deconstruction for sugar release and conversion to biofuels. In this study, suppression of *HCT* in poplars resulted in an increase of H lignin monomer with a commensurate reduction in S lignin monomer. These results accord with those of similar studies, e.g., a large increase in H lignin monomer and strongly reduced S lignin monomer were found when *HCT* was downregulated in transgenic alfalfa plants (Shadle et al., 2007). Similarly, *C3′H* gene downregulation resulted in a significant increase of H lignin monomer at the expense of S lignin monomer in eucalyptus (*Eucalyptus urophylla × E. grandis*) (Sykes et al., 2015).

The *HCT* gene is only involved in the synthesis of S and G monomers and does not include H monomers. Thus, when downregulation of HCT, lignin tends to synthesize H lignin monomers via another pathway (Aymerick et al., 2016). And in this study, *HCT* downregulated poplar tend to produce H lignin monomers at the expense of S lignin monomers. The multibranched structure of the G lignin monomers leads to tight cross-linking between carbohydrates, so that more retention facilitates normal plant growth and increases the strength of the cell wall support (Barsberg et al., 2013). However, lignin rich in S monomers is more likely to depolymerize than lignin rich in G monomers because there are fewer C–C bonds and a higher content of β-O-4 chains that are easier to remove (Ziebell et al., 2016). Therefore, the decrease in S/G ratio in *HCT* transgenic poplars may not benefit the hydrolysis process of lignocellulose. Reduced lignin content in eucalyptus was found to have a much greater effect on reducing recalcitrance than changing the lignin monomers ratio (Sykes et al., 2015). On the whole, the decrease in lignin content and the alteration of lignin structure in *HCT* transgenic poplars favors in reducing the recalcitrance cell walls, but enzyme hydrolysis efficiency and sugar release require further study.

### Fiber Features

The poplars are an important raw material for pulp and paper making in China. Hence, the morphological characteristics of fiber are an important basis for evaluating fiber quality. Studies have shown that thicker and longer fibers are more likely to be resistant to abrasion and breakage. In addition, thin cell walls and large cavity fibers are easy to crush (Wei et al., 2008). Previous studies have reported that reduced lignin content affects fiber quality in transgenic poplars, e.g., Song et al. (2010) discovered that the ratio of fiber length to width in transgenic poplars was reduced following downregulation of the *CCR* gene. *HCT* transgenic poplar has thinner fiber cell walls and larger cell lumens, the characteristic that can make fibers easier to flatten down and become more adhesive (Wu et al., 2013). Fibers isolated from the *HCT* transgenic poplars were longer and wider than those from the CK poplars. These characteristics of the cell structures enable the fiberboards and paper made from *HCT* transgenic poplars to have a relatively high mechanical strength. In general, the fiber characteristics of *HCT* transgenic poplars are more favorable for paper applications.

### Ultrastructure of Cell Wall

Both cellulose MFA and crystallinity are important indicators that affect wood quality. Results of this study have shown that downregulation of *HCT* does not change the orientation of cellulosic microfibrils but increased the cellulose crystallinity of cell walls. Previous studies have also shown that there was no significant difference in the MFA following downregulation of cinnamate 4-hydroxylase (*C4H*) in poplars (Ingela et al., 2010). However, decreases in lignin content and MFA were both revealed in the *Arabidopsis CCR1* mutant, suggesting that different gene regulatory pathways have different effects on MFA (Ruel et al., 2009). It should be noted that the cellulose crystallinity of cell wall in *HCT* poplar has increased (from 35.2 to 39.1%). And, cell wall composition results indicate that the decrease in lignin content lead to a compensatory increase in cellulose content, which is well-accompanied by an increase in crystalline area. Lignin has a mechanically supportive effect on cell walls, and previous studies have demonstrated that reduced lignin content may decrease mechanical stiffness and strength (Özparpucu et al., 2017, 2019). However, the increased cellulose crystallinity is beneficial to improve the physical mechanics of transgenic poplars, including mechanical support. Because, in most woods, the greater the crystallinity, the better the fracture strength and tensile strength of woods (Borrega et al., 2015; Shi et al., 2017).

## Conclusion

The response mechanisms of chemical components and structural properties of wood cell walls to downregulated *HCT* in poplars were revealed. The lignin content of the *HCT* transgenic poplars was decreased with a compensatory increase in cellulose. The cell wall composition changes in the upper part were more obvious than those in the basal part for the *HCT* transgenic poplars. The results of *in situ* observation by Raman technology showed that *HCT* downregulation does not change the distribution of lignin monomers or cellulose in the cell walls. However, the lignin structure changed, and a small intensity of *p*-hydroxyphenyl (H) units was observed, as well as a decrease in the S/G ratio. Compared with the CK poplars, the fiber and vessel cells of the *HCT* transgenic poplars exhibited relatively thin cell walls and bigger lumen diameters. In addition, the fiber length becomes longer. Ultrastructure observations revealed that downregulation of *HCT* does not change the orientation of cellulosic microfibrils, but it led to an increase in the cellulose crystallinity of cell walls.

## Data Availability Statement

The raw data supporting the conclusions of this article will be made available by the authors, without undue reservation.

## Author Contributions

YW and JL designed the project. YW and MS designed the experiments and wrote the manuscript. MS performed the experiments. SZ performed the genomic analysis. YL and MS cultivated the transgenic poplars. All authors read and approved the submitted version of the manuscript.

## Conflict of Interest

The authors declare that the research was conducted in the absence of any commercial or financial relationships that could be construed as a potential conflict of interest.

## Publisher’s Note

All claims expressed in this article are solely those of the authors and do not necessarily represent those of their affiliated organizations, or those of the publisher, the editors and the reviewers. Any product that may be evaluated in this article, or claim that may be made by its manufacturer, is not guaranteed or endorsed by the publisher.
